# Antibiotic prescription monitoring and feedback in primary care in Switzerland: Design and rationale of a nationwide pragmatic randomized controlled trial

**DOI:** 10.1016/j.conctc.2021.100712

**Published:** 2021-01-20

**Authors:** Dominik Glinz, Kimberly A. Mc Cord, Giusi Moffa, Soheila Aghlmandi, Ramon Saccilotto, Andreas Zeller, Andreas F. Widmer, Julia Bielicki, Andreas Kronenberg, Heiner C. Bucher

**Affiliations:** a Basel Institute for Clinical Epidemiology and Biostatistics, Department of Clinical Research, University Hospital Basel, University of Basel, Basel, Switzerland; b Department of Mathematics and Computer Science, University of Basel, Basel, Switzerland; c Centre for Primary Health Care, University of Basel, Basel, Switzerland; d Division of Infectious Diseases and Hospital Epidemiology, University Hospital Basel, Basel, Switzerland; e Infectious Diseases and Vaccinology, University of Basel Children's Hospital, Switzerland; f St. George's University London, London, UK; g Institute for Infectious Diseases, University of Bern, Bern, Switzerland

**Keywords:** Antibiotics, Antimicrobial resistance, Low-cost intervention, Health-system level, Hospitalization, Prescription feedback, Primary care, Routinely collected patient data, Claims, CI, confidence interval, CONSORT, consolidated standards of reporting trials, DRG, Diagnosis Related Groups, EKNZ, Ethikkommission Nordwest-und Zentralschweiz, FMH, Foederatio Medicorum Helveticorum, GP, general practitioners, HRA, Human Research Act, HRO, Human Research Ordinance, RCT, randomized controlled trials, ZSR, Zentralregisternummer

## Abstract

**Introduction:**

Antibiotic consumption is highest in primary care, and antibiotic overuse furthers antimicrobial resistance. In our recently published pilot-RCT, we used monthly aggregated claims data to provide personalized antibiotic prescription feedback to general practitioners (GPs). The pilot-RCT has shown that personalized prescription feedback is a feasible and promising low-cost intervention to reduce antibiotic prescribing. Here, we describe the rationale and design of the follow-up RCT with 3426 GPs in Switzerland. We now have access to pseudonymized patient-level data from routinely collected health insurance data of the three largest health insurers in Switzerland.

**Methods and analysis:**

1713 GPs randomized to the intervention group received once evidence-based treatment guidelines at the beginning, including region-specific antibiotic resistance information from the community and personalized feedback of their antibiotic prescribing, followed by quarterly personalized prescription feedback for two years. The first and the last mailings were sent out in December 2017 and September 2019, respectively. The 1713 GPs randomized to the control group were not notified about the study and they received no guidelines and no prescription feedback. The personalized prescription feedbacks and the analyses of the primary and secondary outcomes are entirely based on pseudonymized patient-level data from routinely collected health insurance data. The primary outcome is prescribed antibiotics per 100 patient consultations during the second year of intervention. The secondary outcomes include antibiotic use during the entire two-year trial period, use of broad-spectrum antibiotics, hospitalization rates (all-cause and infection-related), and antibiotic use in different age groups. If the feedback intervention proves to be efficacious, the intervention could be continued systemwide.

**Ethics and dissemination:**

The trial is publicly funded by the Swiss National Science Foundation (SNSF, grant number 407240_167066). The trial was approved by the ethics committee "Ethikkommission Nordwest-und Zentralschweiz" (EKNZ Project-ID 2017-00888). Results will be disseminated in peer-reviewed journals and international conferences.

## Highlights

• The randomized trial design and blinding assure internal validity.
• The use of routinely collected claims data maximizes the external validity.
• The control group reflects the actual practice of antibiotic prescribing in primary care.
• The primary analysis focuses on the longer-term effect of the feedback intervention.
• The feedback intervention is inexpensive and could be continued systemwide.

## 1 Background

Antibiotic resistance is a serious problem worldwide [1] and directly related to antibiotic consumption [[2], [3], [4]]. In absolute terms, most antibiotics are prescribed in primary care for respiratory and urinary tract infections, which represent the most frequent reasons for physician contact and antibiotic prescribing [[5], [6], [7], [8]].

Strategies to lower antibiotic use in primary care on the system level are difficult to implement. Efficacious strategies to reduce antibiotic prescribing, like face-to-face education or communication training of primary care physicians, are resource-intense, costly, and challenging to apply on a large scale [9,10]. Systemwide feedback interventions on antibiotic prescribing are less resource-intense and low-cost interventions [[11], [12], [13]]. Only a few randomized controlled trials (RCTs) have evaluated feedback interventions for antibiotic prescribing in primary care. These trials have produced inconsistent results. Two trials from the UK, one intervening with a letter from the UK Chief Medical Officier to inform general practitioners (GPs) of practices with the highest 20% on their high prescribing rates [11], and the second trial, sending monthly feedback on antibiotic prescription to GPs for 12 months, both trials lead to moderate reductions in antibiotic prescribing [13].

A trial from Australia found no effect on antibiotic prescription rates from two feedbacks on antibiotics and other drug prescribing that were sent to unselected GPs [14]. In a nationwide trial in Switzerland [12] by our group, the provision of quarterly personalized prescription feedback to primary care physicians over two years did not result in a reduction of antibiotic use (in defined daily doses per 100 consultations) between the intervention and the control group (between-group difference: 0.8% in the first year; 95%CI -2.6%–4.3%; 1.7% in the second year; −5.1%–1.7%), but reduced prescriptions in predefined subgroups of older children and adolescents aged 6–18 years (−8.6% in the first year; 95%CI -14.8% to −1.9%) and younger adults (−4.6% in the second year; 95%CI -7.9% to −1.2%).

Switzerland belongs to the countries with the lowest antibiotic consumption rates in Europe [[15], [16], [17]]. However, prescriptions of antibiotics in primary care are still too high for upper respiratory tract infections, and the high use of quinolone for urinary tract infections is a matter of concern [18]. Also, the emergence of multidrug resistance remains a problem [19].

Here we present the study design of a pragmatic nationwide randomized controlled trial, which assesses a feedback intervention of antibiotic prescribing over two years using patient-level data from routinely collected health insurance claims data. The use of patient-level data will also allow for the monitoring of eventual negative health outcomes like reconsultations and hospitalization. Our intervention targets primary care physicians in Switzerland with average to high antibiotic prescription rates.

## 2 Methods/design

### 2.1 Study design and objective

This is a pragmatic randomized controlled trial in primary care physicians in Switzerland with average to high antibiotic prescription rates. The trial is entirely based on routinely collected individual claims data of the three largest Swiss health insurers (Sanitas, CSS, and Helsana) using pseudonymized identifiers of physicians and patients.

The primary objective is to investigate the effect of a quarterly prescription feedback intervention on the overall antibiotic use in primary care in the second year of intervention (long-term effect). Secondary objectives are to assess effects on the overall antibiotic use within the first 12 months and the entire 24 months intervention duration, and in different age groups. Further secondary objectives address the use of broad-spectrum antibiotics and the impact of the intervention on the eventual unintended effects on hospitalization (all-cause and infection-related) and reconsultation rates.

### 2.2 Inclusion and exclusion criteria

We included and use data from board-certified primary care physicians (Foederatio Medicorum Helveticorum, FMH) in Switzerland with an individual practicing license number "Zentralregisternummer" (ZSR) who are the top 75% prescribers of antibiotic and who have at least 100 patient contacts per year. Group practices sharing a license number are excluded.

### 2.3 Study population, sample size, and power analysis

The data provided by the three health insurers Sanitas, CSS, and Helsana, comprise information on a total of 8422 primary care physicians in Switzerland. These primary care physicians treated at least one patient covered by one of the three health insurances. Due to the usual administrative processing delay of claims data of 6 months by health insurers, the definitive sample size calculation had to be based on claims data from January to December 2016. In this pre-randomization claims data set, we identified 3646 eligible GPs corresponding to the inclusion criteria, as mentioned above. A random sample of 220 physicians from the Basel area was left aside for a different interventional trial. The remaining study population of 3426 physicians were randomized to the intervention or control groups, with 1713 physicians in each arm.

We conducted a power analysis that was based on a trial simulation from 50 bootstrap samples. These bootstrap samples were extracted from the pool of physicians from the database of the year 2016, which was used to select and randomize GPs. The clinically relevant effect that the study aims to detect is, on average 5% expected reduction in prescription rates. Therefore, we simulated a trial for each bootstrap sample and we applied the effect which we expected to observe in the treatment arm. To also account for any variability, we simulated effects from distribution of potential effects, which were "centered" around 5%, for every physician. The standard deviation of the prescription rate in the target population was evaluated based on the data from the year 2016, which was 0.059 on the raw scale and 0.438 on the log scale. Also, we scaled the distribution to account for the expected proportion of non-responders of 15%. For each sample, we then tested the difference using a Mann Whitney *U* test (Wilcoxon rank-sum). Hence, we evaluated the power for each bootstrap sample, and derived medians and confidence intervals from the empirical distribution, which gave an estimated median power of 0.93 (95% CI: 0.90 to 0.96).

### 2.4 Randomization, treatment allocation, and blinding

All 3426 GPs were randomly allocated by a 1:1 ratio to the intervention and control group using R (R Core Team 2017 [20]). GPs in the intervention groups were formally blinded concerning the trial design, and GPs in the control group were fully blinded.

As all RCT relevant data are routinely collected by the automated procession of claims data of health insurances, the outcome assessment may also be considered as formally blinded.

Data analysis will be conducted by a statistician blinded for group assignment of physicians in the trial.

### 2.5 Intervention and control

**Fig. 1:**
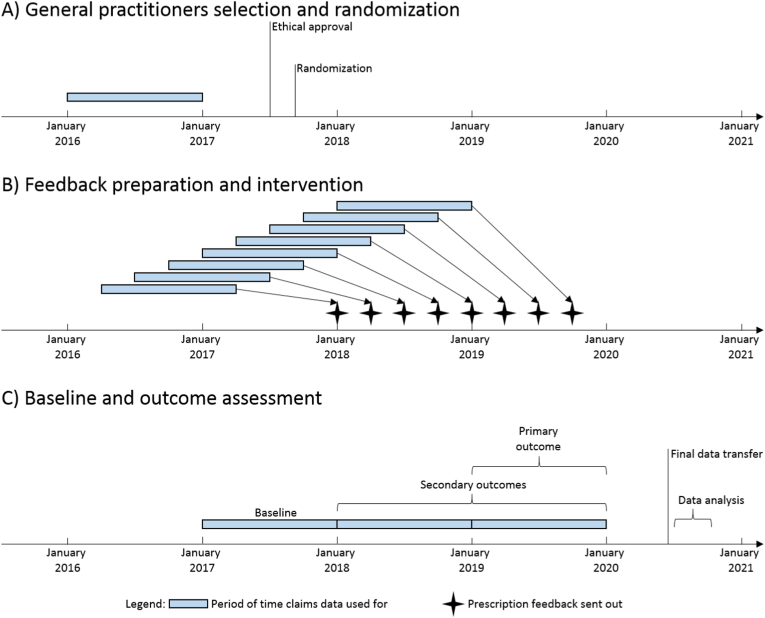
Time periods when claims data were used for general practitioners selection and randomization, for feedback preparation and intervention, and for baseline and outcome assessment.

The first mailing was sent on December 22, 2017 and was followed by seven additional quarterly mailings, with the last mailing sent in late September 2019 (see the timeline in Fig. 1). With the first mailing, all GPs in the intervention group received (1) an accompanying letter explaining the intervention, assuring the full pseudonymization of feedback data, (2) a personalized feedback of antibiotic prescribing; (3) a response card for physicians wishing to opt-out; and (4) evidence-based guidelines on antibiotic prescribing for respiratory and urinary tract infections (see details below). No information on the study design or comparative study purpose was provided.

In the second feedback mailing in April 2018, information on antibiotic resistance data and its regional distribution from the Swiss Centre for Antibiotic Resistance [19] was added.

### 2.6 Personalized antibiotic prescribing feedback

**Fig. 2:**
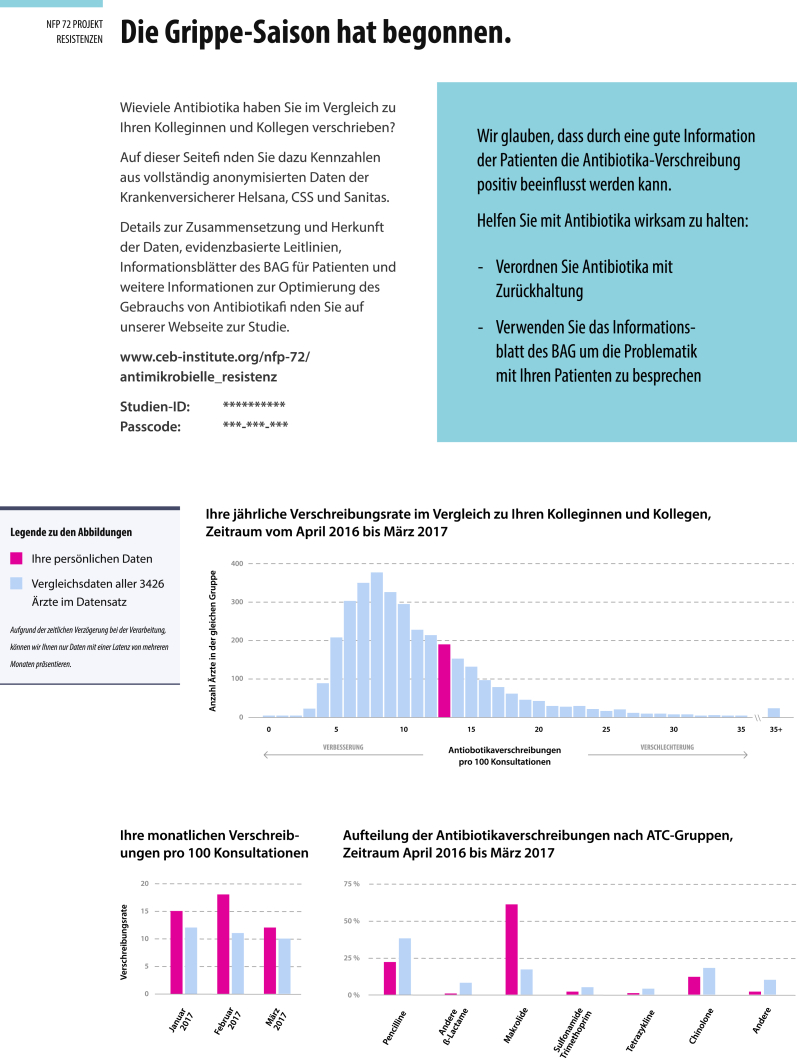
Example of a antibiotic prescribing feedback form.

The personalized antibiotic prescription feedback contained 1) the personal yearly prescription rate (antibiotic prescriptions per 100 consultations) compared with the prescription rates of the peer-physicians, 2) the personal monthly prescription rates for the three months for the same period of the preceding year and its comparison with peer-physicians (the first feedback mailing was based on antibiotic prescription rates between April 2016 to March 2017), 3) the personal yearly prescription rate (antibiotic prescriptions per 100 consultations) stratified by type of antibiotics and compared with the prescription rates of peer-physicians, and 4) a call to action information in a blue box, which varied with each mailing (see example Fig. 2). Personalized antibiotic prescription feedback was provided every three months in the form of a postal mailing and was continuously updated with the most recent complete health insurance data. As there is a six months delay in the health insurance billing records processing and the time needed to prepare the feedbacks, the yearly prescription feedbacks were based on the data from the period of 9 month prior to each sent mailing. All information material and guidelines were also made available to physicians on a password-protected trial website.

### 2.7 Evidence-based guidelines

Evidence-based guidelines for the management of upper and lower respiratory tract and urinary tract infections were provided once with the first mailing to the GPs in the intervention arm. We updated our guidelines from our previous pilot-RCT [12,21] with systematic literature research for additional evidence on the effectiveness of antibiotics for the treatment of these conditions for the time period of 2013 until June 2017. Also, we evaluated the literature on the effectiveness of relevant diagnostic tests for the identification of bacterial-related upper and lower respiratory tract infections in primary care. The guidelines for upper respiratory tract infections were updated together with an expert group by the Swiss Society of Infectious Diseases that is responsible for the development of Swiss national guidelines for the treatment of upper respiratory and urinary tract infections. The guidelines were provided as a paper brochure with the first mailing at the end of December 2017 to all physicians in the intervention group.

Because more than 90% of physicians' offices are located in the German- and French-speaking regions of Switzerland, we provided all information in these two official languages. We assumed that most physicians in the Swiss Italian part know either French or German, since the Swiss educational system includes German or French, and as there is not yet a medical school in the Swiss Italian part of Switzerland.

### 2.8 Outcomes

The primary outcome is the overall antibiotic use, defined as prescribed antibiotics per 100 patient consultations in the second year of intervention lasting from month 13 to month 24 (long-term intervention effect).

The secondary outcomes are (1) overall antibiotic use defined as prescribed antibiotics per 100 patient consultations from month 1–12 (first year of intervention, shorter-term intervention effect); (2) overall antibiotic use defined as prescribed antibiotics per 100 patient consultations over the entire intervention period from month 1 to month 24, with two repeated measurements, over the first and the second 12 month period of intervention; (3) use of broad-spectrum antibiotics (quinolones and oral cephalosporines) per 100 patient consultations; (4) hospitalizations rates (all-cause and related to infections based on Diagnosis Related Groups (DRG) definition); (5) Antibiotic use (prescribed antibiotics per 100 patient consultations) in five specific age groups, in patients 0–14 years, 15–24 years, 25–54 years, 55–64 years, and ≥65 years; and (6) secondary outcomes (3) to (5) will be evaluated separately over the second and first 12 months (from month 13 to month 24 and 1–12 months).

All cause hospitalization will be defined as any hospitalization (DRG-based) within 30 days after a preceeding GP consulation. Infection-related hospitalization will be defined as any hospitalization within 30 days after a preceeding GP consulation with DRG-codes for community acquired pneumonia, urinary sepsis, or sepsis. Reconsultation will be defined as any consultation within 30 days after a first GP consultation or any emergency consultation in a hospital.

### 2.9 Data source and privacy

This RCT is entirely based on routinely collected claims data. The data extracted and used for this trial are provided by the three Swiss health insurers Sanitas, CSS, and Helsana using the physicians' license number ("Zentralregisternummer" i.e. ZSR number). The ZSR number is centrally distributed and provided for all licensed physicians in Switzerland by SASIS/Santésuisse. The information are pseudonymized in the data to ensure confidentiality of records not allowing to identify individual physicians or their patients or the health insurer by the trial investigators. Based on the ZSR numbers, the coordinating data manager from one (Sanitas) of the three health insurers created a unique identifier for each GP. The unique GP identifier allows for the analysis of pseudonymized claims data per physician and per patient, but these identifiers cannot be linked to ZSR numbers by the study investigators or the study staff at any time.

Quarterly prescription feedbacks were prepared by one trial statistician not further involved in the analysis, and printed and packaged by staff not otherwise involved in the trial. Physicians' unique pseudonymous identifiers could be seen in the window of letter envelops containing personalized prescription feedback. The physicians' address was then stuck on the window of the envelope by an independent person not involved in the trial who was in possession of a list of all physician's addresses and the unique pseudonymous identifier. This list was kept on a different password-protected server that could not be accessed by any other person.

The specified study datasets are securely transferred in encrypted format from health insurers to the study server at University Hospital Basel. Study data are stored and processed on infrastructure located within the University Hospital Basel. Data management was conducted in accordance with the procedures used for trial data management by the clinical trial unit. Access to the dataset is strictly limited to the data manager and the statistician of the project.

### 2.10 Claims database

Claims data was formatted by data managers of all three health insurers according to a protocol in a standardized fashion that allowed for data import and the identification of the relevant physician population, the generation of the antibiotic prescribing feedbacks and for the generation of the full claims data set for the final analysis. The relevant variables of claims data can be grouped into five categories, physician identifiers and demographics (Table 1 a)), patient identifier and demographics (Table 1 b)), consultation data including the primary outcome data of antibiotic prescribing (Table 1c)), claims consultation data with relevant diagnostic test ordering, prescribing of all medication (pharmacy costing groups) (Table 1 d)), and hospitalization data (Table 1 e)). Due to the size of the data, only data sets 1a) to 1c), were provided by insurers for the intervention phase (generation of personalized prescribing feedback) of the trial. By the nature of claims data (administrative data), we expected very few missing data, hence, no specific procedure to handle missing data is planned.

**Table 1:** Description of routinely collected data – basic information of primary care physician and consulations

Variable groups	Description of variables
Table 1a) Physician identifier and demographics	
Physician identifier	Encrypted number (pseudonymized ZSR number)
Canton	Location of the practice (Swiss cantons)
Speciality	General internal medicine, pediatrics or family doctor
Table 1b) patient identifier and demographics	
Patient Identifier	Encrypted number (pseudonymized from health insurerdatabases)
Age category (in 2017)	For years 0–5; 6–10; 11–15; in 5-year categories up to registered maximum age
Majority	Yes/no
Gender	Male, female
Insurance membership Identifier	Pseudonymized codes for the three health insurers
Table 1c) consultation data including the primary outcome data of antibiotic prescribing	
Consultation	Date of consultation, specific Tarmed code(s) and total number of Tarmed code(s)
Table 1d) claims consultation data with relevant diagnostic test ordering, prescribing of all medication (pharmacy costing groups)	
Diagnostic	Date of laboratory analysis, code(s) from the list of laboratory analyses
Pharmacy-Cost groups	Date of the prescription, complete ATC code, total number of prescriptions in ATC group, total costs according to claims in CHF, costs per package
Antibiotics	Date of the prescription, all pharma codes from ATC group J01, complete ATC code (ATC group J01), number of prescribed packages, total costs according to claims in CHF, costs per packages in CHF
Table 1e) Inpatient information	
Hospitalization	Date of hospital admission, date of hospital discharge, DRG code(s)

### 2.11 Data analysis

Statistical analysis will be performed by the trial statistician using R (R Core Team 2017 [20]). All analyses will be conducted using the final dataset, which will be available in September 2020 (see also timeline in Fig. 1C) and will follow CONSORT guidelines and intention-to-treat principles [[22], [23], [24]]. A flowchart will describe the inclusion and follow-up of GPs by study arm. Baseline characteristics will be described by study arm with summary statistics such as median and interquartile range or number and percentage; no formal testing between arms will be performed [25]. Outcomes will be described by arm using summary statistics.

The primary outcome, the proportion of individuals with an antibiotic prescription in the second year of the intervention, will be assessed by ANCOVA modeling, with the outcome as a response, intervention (yes/no) as a factor of interest, and baseline year antibiotic prescription rate as a covariate (the baseline year is January–December 2017). Other baseline covariates of interest include comorbidities (based on pharmacy cost groups) patients' age, annual patient volume (total number of consultations per GP), and seasonality. For the primary outcome analysis, the coefficient estimates and their 95% CI will be reported. Secondary outcomes will be evaluated in the same way.

Effects for the period month 1 to month 24 will be modeled using a linear mixed model including the intervention (yes/no), time (baseline, first year, second year), an interaction term for the intervention with time, and by treating physician as random effects. Mean percentage changes from baseline will be derived for the intervention and control groups. Other baseline covariates of interest will be included as for the primary analysis.

The rates of hospitalizations and reconsultations will be modeled using logistic regressions including the intervention as a factor of interest and other relevant covariates. Hospitalization rates in the intervention and control group, with exact 95% CIs will be reported, and odds ratios for the intervention arm vs. control will be derived from the coefficients estimated for the logistic regression model.

The analysis in specific age groups will be similar to the primary analysis over the respective periods and stratified by age groups i) 0–14 years, ii) 15–24 years, iii) 25–54 years, iv) 55–64 years, and v) 65 years and over.

### 2.12 Ethics and dissemination

The study trial protocol was approved by the lead ethics committee Ethikkommission Nordwest-und Zentralschweiz (EKNZ Project-ID 2017-00888) according to the simplified approval procedure based on Art. 34 "Absence of informed consent" of the Human Research Act (HRA) and based on Art. 37–40 "Use of Biological Material and Health-Related Personal Data for Research in the Absence of Informed Consent" of the Human Research Ordinance (HRO). Without further requests by the lead ethics committee, the approval of the lead ethics committee is valid for all remaining cantons of Switzerland.

## 3 Discussion

To our knowledge, this is the first pragmatic RCT using routinely collected patient level claims data to assess the effects of quarterly personalized prescription feedback on antibiotic prescribing in primary care on the national health system level. In our pilot RCT [12,21], feedbacks and final analyses were based on monthly aggregated claims data, and not on patient-level data. As a consequence, the analyses, including subgroups, were limited because of the aggregated nature of the data. Built on the experience of the pilot RCT, we planned the present trial protocol using patient-level data from routinely collected claims data. For instance, a more comprehensive quarterly feedback to GPs (prescribing rates instead of defined daily doses) and the patient-level data will allow for more detailed analyses of antibiotic prescribing patterns and to correlate them for example with test ordering like CRP, pharyngeal swabs or urine cultures.

Moreover, patient-level data allows for investigating multiple antibiotic prescribing in the same patient and the analysis of potentially unintended consequences of the intervention like hospitalization, emergency ward consultations, and reconsultations due to untreated bacterial infections. We will also receive detailed data on ordering of diagnostic laboratory tests, X-rays and referals to specialists that are relevant for the management of respiratory and urinary tract infections. This will allow for secondary analysis of physician practice patterns and antibiotic prescribing and provide useful data for the planned health economic analyses.

## 3.1 Strengths and limitations

Our pragmatic trial design will allow to generate evidence of high internal and external validity on the effectiveness of antibiotic prescribing feedback in primary care, due to the randomized trial design and blinding for the nature of the intervention, and the use of routinely collected claims data, which assures continuous and complete follow-up data with no interference with daily routine for data collection. Full blinding of the control group by use of Zelen's design [26] allows for a comparator that reflects the actual practice of antibiotic prescribing in primary care in Switzerland at large. If our feedback intervention proves to be efficacious, the intervention could be continued systemwide as the infrastructure has been established for the purpose of this trial. This would allow continuous monitoring of antibiotic prescribing at low costs and to intervene if peer-based prescribing benchmarks are constantly overrun by some GPs.

## Declarations

Ethics approval and consent to participate.

The study trial protocol was approved by the lead ethics committee Ethikkommission Nordwest-und Zentralschweiz (EKNZ Project-ID 2017-00888) according to the simplified approval procedure based on Art. 34 "Absence of informed consent" of the Human Research Act (HRA) and based on Art. 37–40 "Use of Biological Material and Health-Related Personal Data for Research in the Absence of Informed Consent" of the Human Research Ordinance (HRO). Without further requests by the lead ethics committee, the approval of the lead ethics committee is valid for all remaining cantons of Switzerland.

## Patient and public involvement

It was not appropriate or possible to involve patients or the public in the design, or conduct, or reporting, or dissemination plans of our research.

## Consent for publication

Not applicable.

## Availability of data and materials

No additional data to be shared.

## Funding

Supported by the 10.13039/100000001Swiss National Science Foundation (407240_167066). The funders had no role in design and conduct of the study; collection, management, analysis, and interpretation of the data; and preparation, review, or approval of the manuscript or its submission for publication.

## Author contributions

AW, AZ, RS and HCB conceived the study and developed the protocol. GM, RS and HCB created the personalized feedback system. AW, AZ and HCB wrote the clinical guidelines. GM, RS and SA managed the data. DG, GM, RS and SA coordinated the set-up of the postal feedback intervention. GM and SA analyzed the data. DG wrote the first draft and all authors made revisions on the manuscript. All authors read and approved the final version of the manuscript. HCB is the guarantor.

## Declaration of competing interest

DG, KAM, GM, SA, RS, AZ, AW, JB, AK and HCB declare no conflict of interest. The Basel Institute for Clinical Epidemiology and Biostatistics, University Hospital Basel, Basel, Switzerland was supported in 2013 by Santésuisse, the umbrella association of Swiss social health insurers. DG, KAM, GM, SA, RS, AZ, AW, JB, AK and HCB declare no other financial relationships with any organization that might have an interest in the submitted work in the previous 3 years and no other relationships or activities that could appear to have influenced the submitted work.
